# Autophagy is induced and modulated by cholesterol depletion through transcription of autophagy-related genes and attenuation of flux

**DOI:** 10.1038/s41420-021-00718-3

**Published:** 2021-10-29

**Authors:** Keren E. Shapira, Guy Shapira, Eran Schmukler, Metsada Pasmanik-Chor, Noam Shomron, Ronit Pinkas-Kramarski, Yoav I. Henis, Marcelo Ehrlich

**Affiliations:** 1 School of Neurobiology, Biochemistry and Biophysics, George S. Wise Faculty of Life Sciences, Tel Aviv University, Tel Aviv, 6997801 Israel; 2 Sackler Faculty of Medicine, Tel Aviv University, Tel Aviv, 6997801 Israel; 3 Edmond J Safra Center for Bioinformatics, Tel Aviv University, Tel Aviv, 6997801 Israel; 4 Bioinformatics Unit, George S. Wise Faculty of Life Science, Tel Aviv University, Tel Aviv, 6997801 Israel; 5 Shmunis School of Biomedicine and Cancer Research, George S. Wise Faculty of Life Sciences, Tel Aviv University, Tel Aviv, 6997801 Israel

**Keywords:** Autophagy, Lipids

## Abstract

Perturbations to cellular homeostasis, including reduction of the cholesterol level, induce autophagy, a self-digestion process of cellular constituents through an autophagosomal–lysosomal pathway. In accord with its function as a membrane organizer and metabolic sentinel, the cellular response to cholesterol depletion comprises multiple phenomena, including the activation of transcriptional responses, accumulation of reactive oxygen species (ROS), and activation of stress-related signaling pathways. However, the molecular mechanisms by which cholesterol depletion regulates autophagy and the putative involvement of transcriptional responses, ROS and/or stress-related signaling in autophagy regulation in this biological context are not fully understood. Here, we find that cholesterol depletion regulates autophagy at three different levels. First, employing RNA-seq, we show that cholesterol depletion increases the expression of autophagy-related genes independent of ROS or JNK activity. Second, analysis of LC3 lipidation and intracellular localization, and of p62 levels and degradation kinetics, reveals that cholesterol depletion mediates autophagy induction while interfering with autophagic flux. Of note, only the latter depends on ROS accumulation and JNK activity. In view of the common use of cholesterol-reducing drugs as therapeutic agents, our findings have important implications for multiple cellular settings in which autophagy plays a prominent role.

## Introduction

Cholesterol is a plasma membrane organizer, which plays a major role in determining the molecular properties of cell membranes [1], including the formation of cholesterol/sphingolipid-enriched domains [2–6]. Cholesterol homeostasis is under regulation by several mechanisms, which modulate sterol synthesis, uptake, and turnover [7, 8]; its disturbance is linked to various diseases, from atherosclerosis and cardiovascular illnesses to neurodegenerative diseases and cancer [9–12]. Thus, inhibition of HMG-CoA reductase by statins is a major therapeutic approach for treatment of multiple diseases [13, 14]. Such treatments evoke multi-layered cellular responses aimed at restoring cholesterol homeostasis [15]. Statin-mediated reduction of cholesterol can also affect the transcription and translation of signaling mediators, as we have demonstrated for transforming growth factor-β (TGF-β) activated Smad signaling involving overactivation of double-stranded RNA-dependent protein kinase and c-Jun N-terminal kinase (JNK), culminating in augmented Smad3-mediated responses [16].

Autophagy is a self-digestion process of cellular constituents via an autophagosomal–lysosomal pathway. It provides a mechanism to eliminate damaged proteins, toxic protein aggregates, whole organelles and invading pathogens, and functions in catabolic quality control and recycling of cellular components under nutrient-limiting conditions [17–21]. Autophagy is comprised of several consecutive events: induction and nucleation of the autophagosome, cargo sequestration, delivery and fusion with the lysosome, degradation of the autophagosome with its cargo, and recycling of the degraded material [22]. Autophagy induction is negatively regulated by mTOR1, while autophagosome formation and elongation are regulated by several autophagy-related (Atg) proteins [23]. These steps involve two ubiquitin-like conjugation systems (including lipidation of Atg8/LC3 protein) and a class III PI3K complex. The delivery and fusion of the autophagosome with the lysosome are mediated by SNARE and Rab7. Finally, after cargo breakdown in the autolysosome, building blocks are recycled to the cytosol [23]. Completion of the degradation and recycling steps defines autophagic flux. In selective autophagy, the induction signal is generated by the targeted cytoplasmic component, followed by recruitment of the core autophagic machinery. Thus, damaged organelles or proteins cargoes are ubiquitinated and recognized by autophagy proteins acting as ubiquitin receptors (e.g., p62/*SQSTM1*), which link them to the autophagy machinery by binding the lipidated Atg8/LC3 on the forming autophagosome [24–27]. Both p62/*SQSTM1* and lipidated-LC3 (LC3-II) serve as autophagy markers.

A potential link between autophagy and cholesterol was suggested by the induction of autophagy following statin treatment [28], the increased expression of autophagy genes following cholesterol reduction [29], and the effects of cholesterol on autophagosome transport and fusion with the lysosome [30, 31]. While the molecular mechanisms by which cholesterol depletion (CD) regulates autophagy remain unknown, statin treatment induces reactive oxygen species (ROS) formation, which can regulate autophagy [32–41]. However, how ROS regulates autophagy in cholesterol-depleted cells is yet unknown. Here, we studied the effects of CD on the expression of autophagy-related genes, autophagy induction, and autophagic flux. Our results reveal compound effects of CD on different aspects of autophagy, which depend partially on statin-induced ROS accumulation. Thus, reduced cholesterol levels enhance expression of autophagy-related genes and induce autophagy independent of ROS. Yet, CD concomitantly attenuates autophagic flux in a ROS- and JNK-dependent manner.

## Results

### Cholesterol depletion increases expression of autophagy-related genes

**Fig. 1:**
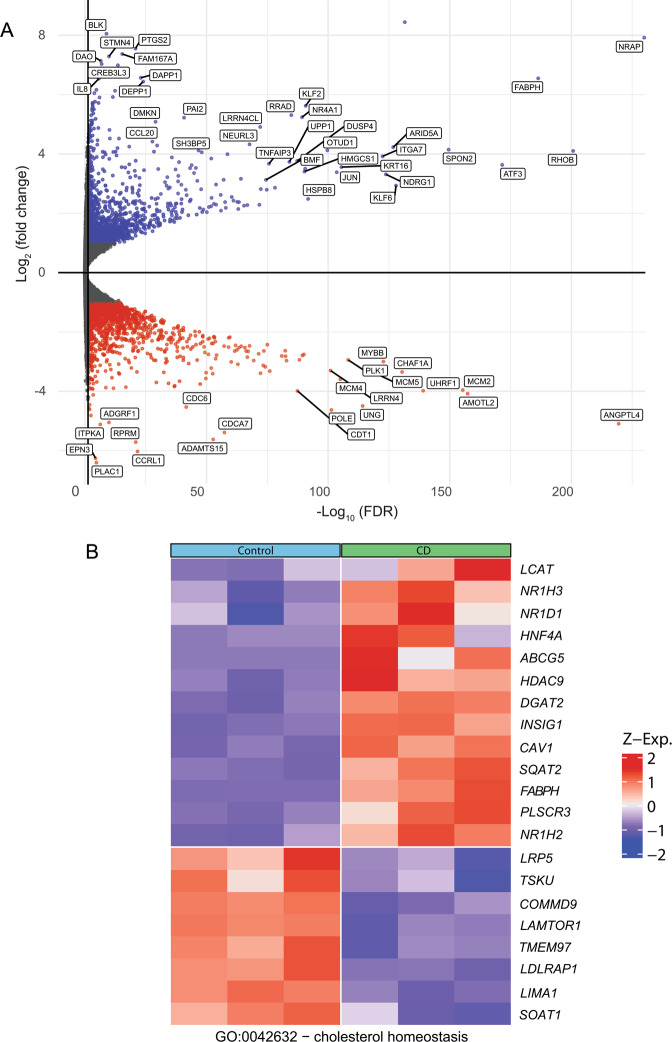
Cholesterol depletion alters mRNA levels of a large number of genes, including cholesterol homeostasis genes. Mv1Lu cells were treated (or not) by statin in the presence of mevalonate and LPDS (16 h) to reduce their cholesterol level, and then subjected to RNA-Seq (see “Materials and methods”). **A** Volcano plot depicts genes with significantly increased or reduced expression levels following CD relative to untreated cells. **B** Heat map of differentially expressed genes related to cholesterol homeostasis. Only those genes with significant differential expression (FDR < 0.05) are shown.

**Fig. 2:**
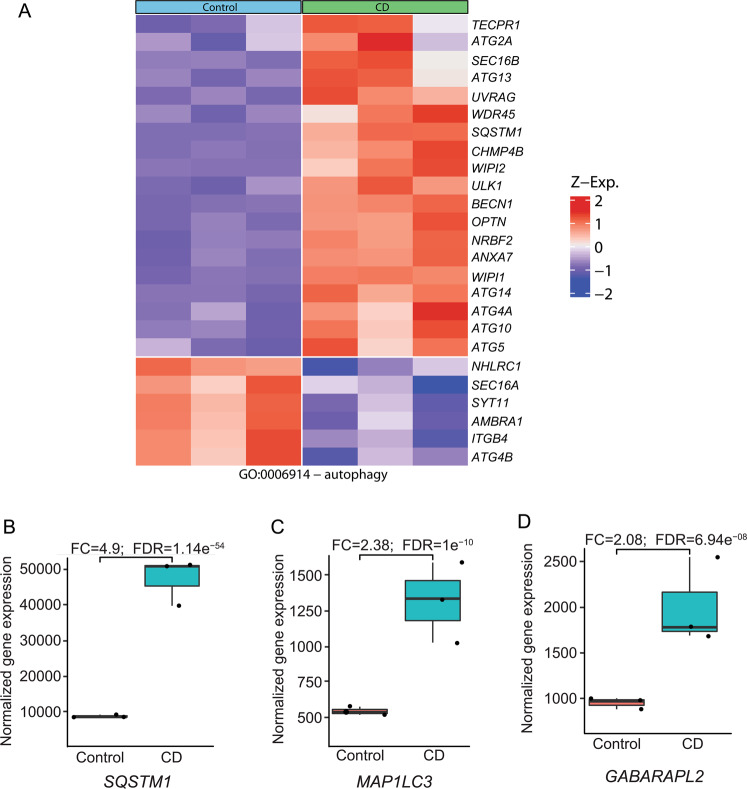
Cholesterol depletion increases the mRNA levels of multiple autophagy-related genes. RNA-seq of Mv1Lu cells was as in Fig. 1. **A** Heat map of differentially expressed genes related to autophagy. Only those genes with significant differential expression (FDR < 0.05) are shown. **B**–**D** Box plot of three specific autophagy-related genes whose expression was significantly enhanced by CD: *SQSTM1*, which encodes p62 (**B**), *MAP1LC3*, encoding LC3 (**C**), and *GABARAPL2*, which encodes GEF-2 (**D**). The fold change (FC) and FDR-adjusted *P*-values are indicated.

To test the transcriptomic response to prolonged reduction in the cholesterol level, we subjected Mv1Lu cells to CD (16 h, with lovastatin and mevalonate in the presence of lipoprotein deficient serum (LPDS)), reducing the free cholesterol level by ~30% [16]. These cells and conditions were chosen as we have recently demonstrated that they induce marked alterations to the expression and activation of several signaling mediators [16]. The gene expression profiles of the treated and untreated cells were compared using RNA sequencing (RNA-seq). A volcano-plot analysis demonstrated comparable numbers of up- and downregulated genes, suggesting that the induced changes are specific and are not general effects on transcription (Fig. 1A). Due to the large number of transcripts presenting significantly altered expression upon CD, and in order to visualize the relative weight of the cellular processes or intracellular compartments to which these transcripts belong, we generated Revigo TreeMaps (see Supplemental Materials and Methods) of GO enrichment (Supplementary Figs. 1–3). Shown here are Treemaps depicting “Upregulated Cellular Processes” (Supplementary Fig. 1), “Downregulated Cellular Processes” (Supplementary Fig. 2), and “Upregulated Cellular Compartments” (Supplementary Fig. 3). In accord with the large number of altered transcripts (Supplementary Table 1), multiple categories are observed in all three maps. In these supplementary figure maps, Cholesterol biosynthetic processes (yellow) and autophagosome assembly (green) were among the GO terms appearing in the analysis of upregulated transcripts, while the cell compartment analysis revealed phagocytic vesicle (comprising also autophagosome) as the main GO in the cellular compartments upregulated transcripts map. Given the role of cholesterol as a metabolic sentinel and the involvement of sterol regulatory element-binding proteins (SREBPs) in the expression of autophagy-related genes [29], we identified which cholesterol metabolism (Fig. 1B) or autophagy-related genes (Fig. 2A) were affected. Notably, we identified a panel of autophagy-related genes whose expression significantly increased (Fig. 2A). KEGG analysis of the autophagy pathway indicated a distribution of autophagy-promoting genes induced by CD to multiple steps of this process (Supplementary Fig. 4). Three of the autophagy-related genes whose expression increased upon CD (*MAP1LC3A*, *SQSTM1*, *GABARAPL2*), involved in different stages of autophagic flux (elongation, specific cargo selection, and autophagosome maturation) [27, 42], were selected for calculation of the significance of the change in expression (Fig. 2B–D), yielding highly significant differences (FDRs of 10^−8^ to 10^−54^).

### Cholesterol depletion induces autophagy

**Fig. 3:**
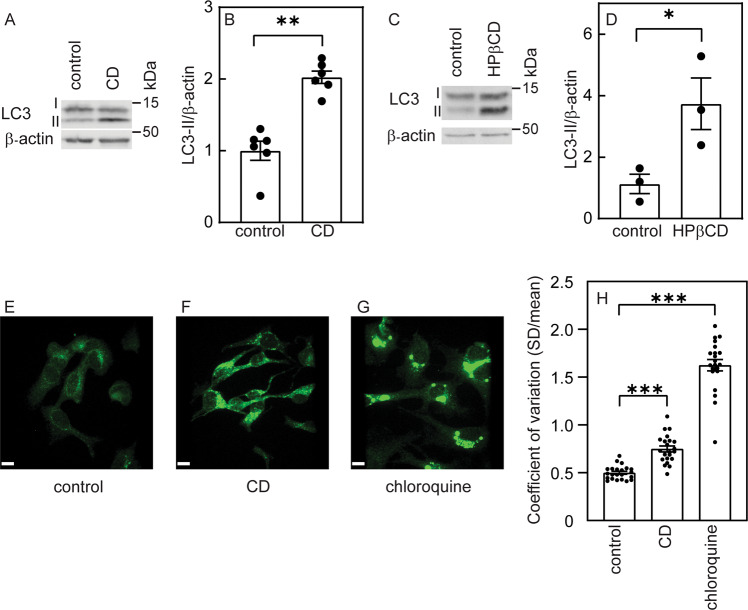
Cholesterol depletion induces LC3-II accumulation. Mv1Lu cells grown in 35 mm dishes were subjected (or not) to CD by statin (**A**, **B**) or by HPβCD (**C**, **D**) as described under “Materials and methods”. After 16 h, they were lysed and analyzed by immunoblotting for LC3 (autophagy marker) and β-actin (loading control). The bands were quantified by ECL (see “Materials and methods”). **A**, **C** Representative blots. The levels of LC3-II (the lower band, which represents the lipidated protein) were elevated following CD by either statin (**A**) or HPβCD treatment (**C**). **B**, **D** Quantification of LC3-II levels relative to β-actin. The level measured for the control sample (untreated cells) was taken as 1. Bars are mean ± SEM of 6 (**B**) or 3 (**D**) independent experiments (*P < 0.05; **P < 0.01; Student’s two-tailed *t*-test). **E**–**G** LC3 punc*t*a accumulation in cholesterol-depleted cells. Bar, 10 μm. Cells were either not treated (control; **E**), cholesterol-depleted by statin (**F**), or treated by chloroquine (10 μM) together with the CD treatment, fixed and stained with rabbit anti-LC3B antibody followed by Alexa 488-goat anti-rabbit IgG and subjected to confocal microscopy. Representative fluorescent images of an experiment conducted three times with similar results are shown. **H** Quantification of the LC3 accumulation by immunofluorescence. Cells were visualized by spinning disk confocal microscopy and analyzed for the distribution of LC3 fluorescent labeling. Data are presented as the coefficient of variation under each condition. Bars are mean ± SEM of measurements on 25 (control and CD) or 23 (chloroquine) cells. Both CD and chloroquine treatments significantly increased the coefficient of variation of the fluorescence intensity distribution (***P < 0.001; Student’s two-tailed *t*-test).

Next, we tested whether lovastatin-mediated CD induces autophagy in Mv1Lu cells. LC3 levels (LC3-I and its lipidated form, LC3-II) were measured by immunoblotting in cells incubated for 16 h in growth medium (control) or in cells subjected to CD by lovastatin and mevalonate (16 h). Figure 3A, B demonstrates that CD-mediated accumulation of LC3-II, indicative of autophagy induction. To exclude involvement of side effects of the statin treatment, we reduced cholesterol to a similar level [16] with 2-hydroxypropyl-β-cyclodextrin (HPβCD; 15 mM, 16 h) (Fig. 3C, D), resulting in a similar effect on LC3-II accumulation. Next, we probed for the intracellular distribution of LC3, as LC3 puncta accumulation is typical of autophagy induction [23]. Indeed, confocal fluorescence microscopy revealed a significant increase in LC3-positive puncta following CD (Fig. 3E–H). Chloroquine treatment, which blocks the binding of autophagosomes to lysosomes and lysosomal degradation, thus increasing LC3-positive puncta, mediated an even higher effect (Fig. 3G, H).

**Fig. 4:**
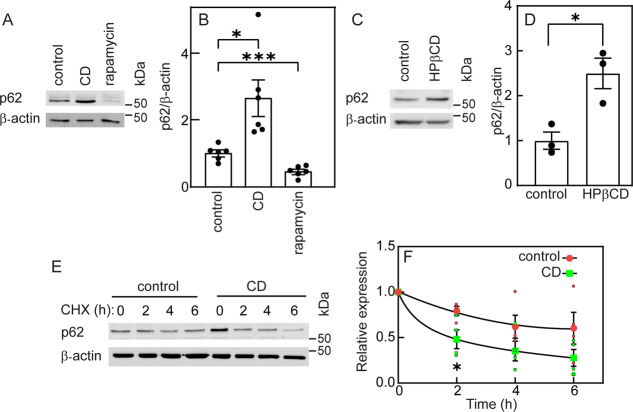
p62 expression level is increased following cholesterol depletion. Mv1Lu cells were grown and treated (or not) by statin or by HPβCD to induce CD as in Fig. 3, or treated with rapamycin (150 nM, 16 h). They were then lysed and analyzed by immunoblotting for p62 and β-actin (loading control). **A**, **C** Representative blots of the effects of statin-mediated CD and of rapamycin (**A**), or of HPβCD-induced CD, on p62 levels (**C**). **B**, **D** Quantification of p62 levels relative to β-actin. The level of p62 in the control sample for each blot was taken as 1. Bars are mean ± SEM of 6 (**B**) or 3 (**D**) independent experiments. Both CD treatments induced a significant increase in the p62 level relative to untreated cells, while rapamycin reduced the p62 level (*P < 0.05; ***P < 0.001; Student’s two-tailed *t*-test). **E** A representative blot of p62 degradation. Cells were subjected (or not) to statin-mediated CD, serum-starved (2 h), and subjected to CHX-chase degradation assay (“Materials and methods”). Note that the zero time point of CHX addition is after 16 h of CD treatment. **F** Quantification of p62 degradation (mean ± SEM, *n* = 4 independent experiments). Data were normalized to β-actin, taking the zero time (prior to CHX addition) for each experimental condition as 1. The degradation rate of p62 was significantly enhanced following CD (*P < 0.05, Student’s two-tailed *t*-test, comparing between control and CD samples at the same time point).

Another hallmark of autophagy is degradation of the selective autophagy receptor, p62 [23]. CD mediated by statin or HPβCD (Fig. 4 resulted in increased p62 levels. In contrast, rapamycin treatment (which induces autophagy *via* mTOR inhibition) reduced p62. Given the apparent discrepancy between the expected reduction in p62 in cells undergoing autophagy and the observed p62 accumulation following CD, we measured p62 degradation following cycloheximide (CHX) chase (Fig. 4E, F). Cells were cholesterol depleted (or not) for 16 h, followed by inhibition of protein synthesis with CHX and measurement of p62 levels as a function of time (Fig. 4E, F). This revealed enhanced degradation kinetics of p62, in accord with the expectation in cells undergoing autophagy.

**Fig. 5:**
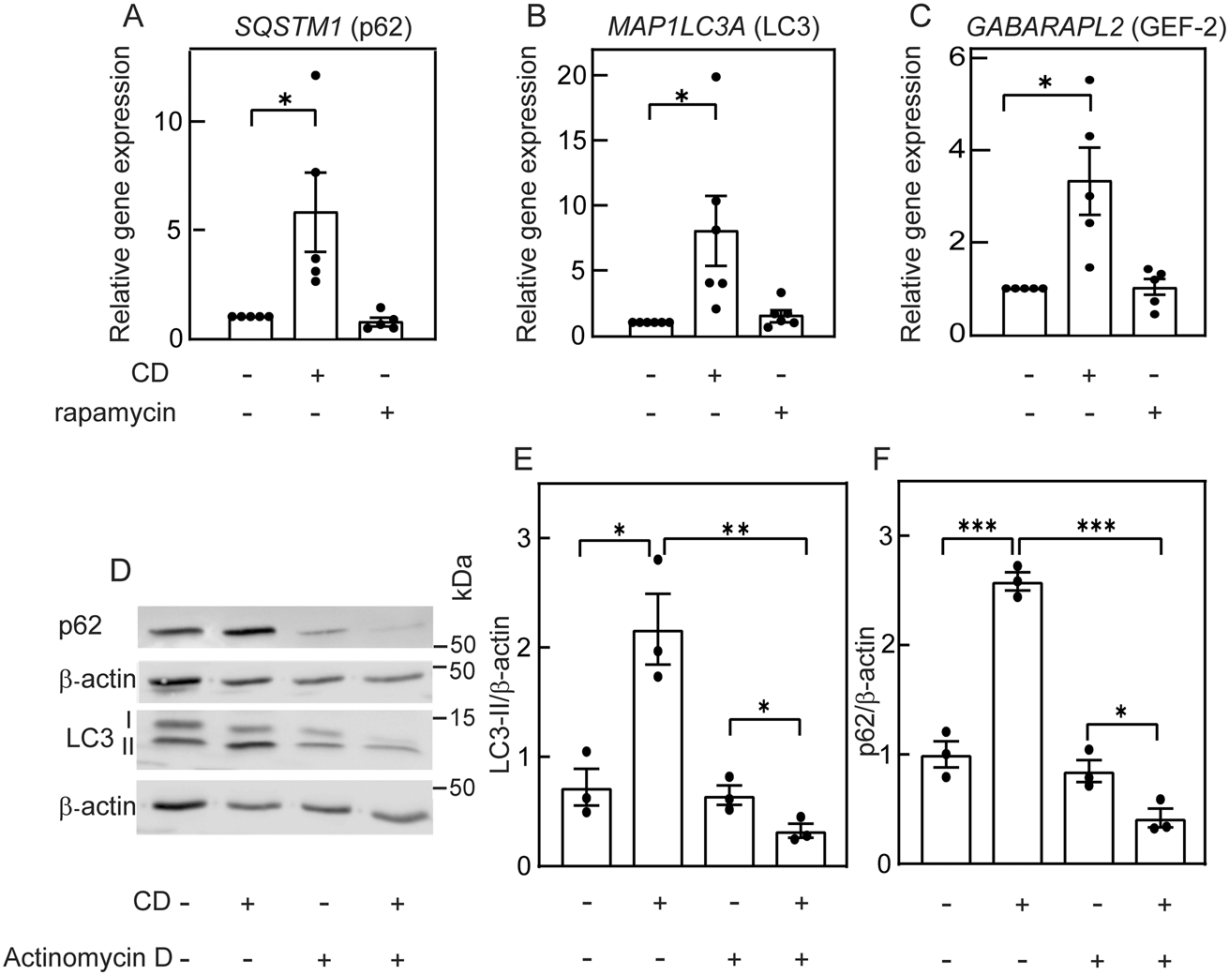
Cholesterol depletion-mediated increase in mRNA levels of autophagy markers is not essential for autophagy induction. **A**–**C** RT-qPCR analysis of **A** *SQSTM1* (encodes for p62); **B** *MAP1LC3* (encodes for LC3); and **C** *GABARAPL2* (encodes for GEF-2). Mv1Lu cells were treated (or not) for CD by statin or with rapamycin as in Fig. 4. RT-qPCR was as described under “Materials and methods”. Data (mean ± SEM; 5 or 6 independent experiments in each case) were normalized to the cDNA levels of β-actin, taking the level of the respective mRNA in untreated cells (control) as 1. The mRNA levels were significantly elevated in cholesterol-depleted cells, but were not affected by rapamycin treatment (*P < 0.05, Student’s two-tailed *t*-test). **D**–**F** Induction of autophagy by CD persists in the presence of actinomycin D. Cells were treated (or not) for CD by statin in the absence or presence of actinomycin D (1 μg/ml, added together with the statin). After 16 h, cells were lysed and analyzed by immunoblotting for LC3 and p62, using β-actin as loading control. **D** A representative blot. **E**, **F** Quantification of LC3-II (**E**) and p62 (**F**) levels relative to β-actin. The level measured for the control sample (untreated cells) was taken as 1. Bars are mean ± SEM of 4 independent experiments (*P < 0.05; ***P < 0.001; Student’s two-tailed *t*-test).

The accumulation of p62 on the one hand and its enhanced degradation on the other are seemingly in contradiction. Such a situation may arise from a combination of enhanced expression and accelerated degradation. To test this hypothesis, we measured the transcript levels of *SQSTM1* (encoding p62), *MAP1LC3A* (LC3), and *GABARAPL2* (GEF-2) in control cells, cholesterol-depleted cells, or rapamycin-treated cells. Figure 5A−C shows that CD induced significantly higher levels of *SQSTM1* (p62) mRNA, as well as of the two other autophagy-related genes. In contrast, no such increase was detected with rapamycin. To further test whether transcription has a role in the altered levels of LC3-II or p62 induced by CD, we studied the effect of adding the transcription inhibitor actinomycin D to the CD treatment (Fig. 5D–F). This combination significantly altered the LC3 expression pattern (Fig. 5D); LC3-I was drastically reduced, while LC3-II remained at a level similar to cells treated with actinomycin D alone, as observed upon autophagy induction [23]. Importantly, comparison of LC3-II levels in cells treated by CD alone and by CD plus actinomycin D revealed a significant reduction of LC3-II under the latter conditions (Fig. 5E). A similar reduction was observed for p62 levels under the above conditions (Fig. 5F), in accord with an autophagic process. Taken together, our results indicate that CD induces two phenomena: (i) increased expression of autophagy-related genes, as shown by RNA-seq (Figs. 1 and 2) and real-time quantitative reverse transcriptase-PCR (RT-qPCR) (Fig. 5A–C); (ii) induction of autophagy, as shown by accumulation of LC3-II (Fig. 3A–D), formation of LC3 puncta (Fig. 3E, F), and enhanced p62 degradation (Fig. 4). The combined effect of these two phenomena is the accumulation of p62 proteins in cholesterol-depleted cells, unlike the effect of rapamycin, where *SQSTM1* expression is not enhanced, and p62 protein expression is reduced. Of note, in the absence of transcription (CD in the presence of actinomycin D), the increase in p62 was eliminated, revealing an autophagy-dependent reduction in its level and suggesting that autophagy mediated by CD can proceed without transcription.

### ROS and JNK alter flux in autophagy mediated by cholesterol depletion

**Fig. 6:**
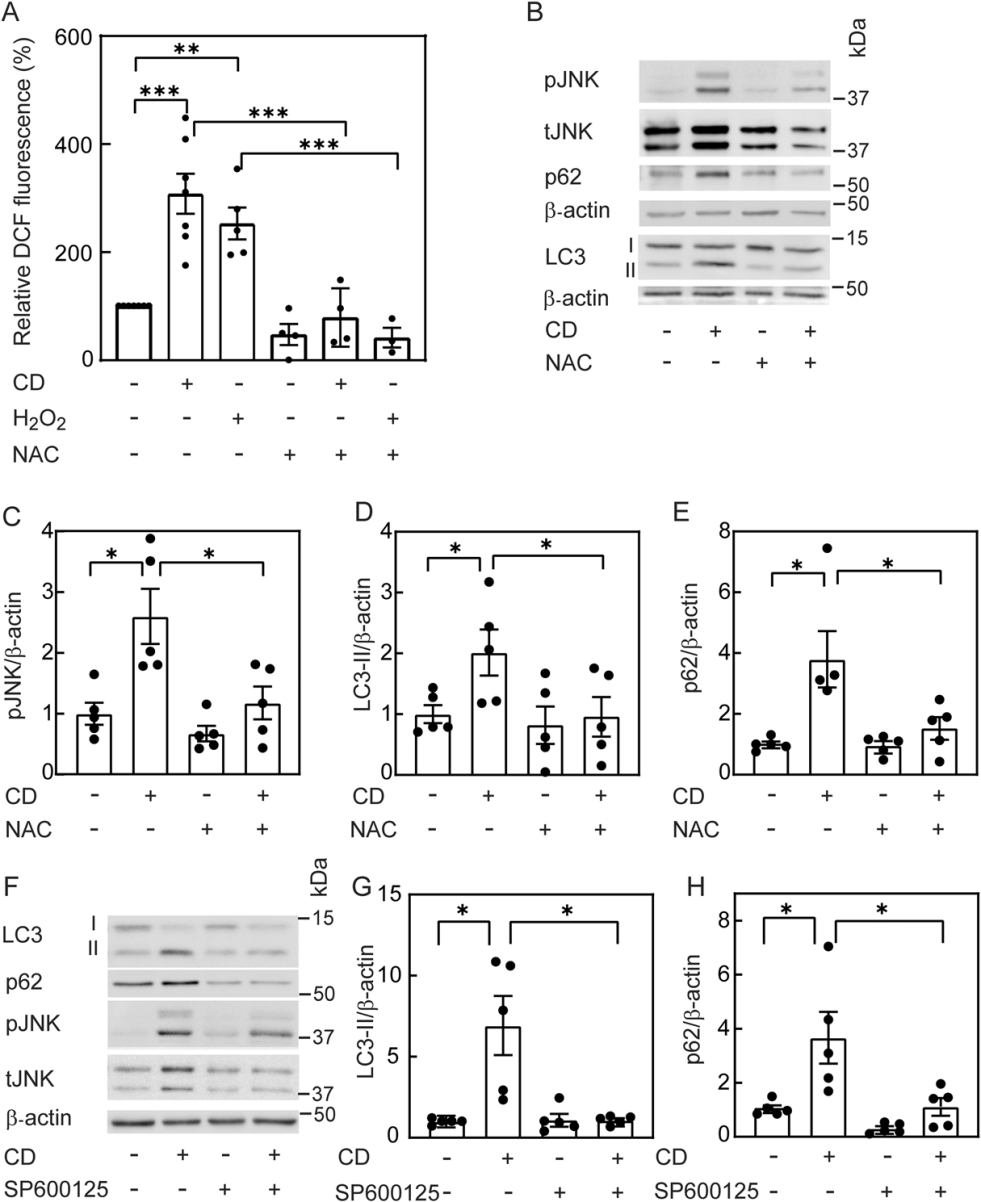
Elevation of ROS by cholesterol depletion is required for JNK activation and for the effects on LC3-II and p62 levels. Mv1Lu cells in 6-well plates were subjected (or not) to CD by statin (16 h) with or without the ROS scavenger NAC (10 mM; **A**–**E**), or the JNK inhibitor SP600125 (20 μM; **F**–**H**). **A** CD elevates ROS. Cells were treated (or not) with H_2_O_2_ (0.75 mM, 2 h; positive control), and incubated at 37 °C with DCF (10 μM, 30 min). After washing, DCF fluorescence intensity was determined by FACS with excitation at 485 nm and emission at 525 nm, taking the intensity measured in untreated cells in the same experiment as 100%. Bars are mean ± SEM of 3–7 independent experiments. Asterisks indicate a significant difference between the indicated pairs (**P < 0.01; ***P < 0.001; one-way ANOVA and Bonferroni post hoc test). **B**–**E** The ROS scavenger NAC prevents the effects of CD on JNK activation and on LC3-II and p62 levels. A representative blot is shown in panel **B**. Quantification of the effect of NAC on the levels of the proteins tested is depicted for pJNK (**C**), LC3-II (**D**), and p62 (**E**). The control sample (no CD, no NAC) for each protein was taken as 1. Bars are mean ± SEM of 5 independent experiments (*P < 0.05; Student’s two-tailed *t*-test). **F**–**H** Inhibition of JNK ac*t*ivity prevents the effect of CD on LC3-II and p62 levels. **F** A representative blot. **G**, **H** Quantification of the effects on LC3-II and p62 protein levels. Bars, mean ± SEM of 5 independent experiments. In each blot, the untreated sample (control) was taken as 1. Asterisks indicate significant differences between the pairs of samples indicated by the brackets (*P < 0.05; Student’s two-tailed *t*-test).

Statin treatment induces ROS formation in different cell types [32, 33], and ROS may regulate autophagy through diverse molecular mechanisms [34–41]. Moreover, ROS and CD activate JNK [16, 43], which regulates autophagy [36, 44–48]. Therefore, we proceeded to examine the involvement of ROS and JNK in autophagy induced by CD. To explore whether CD induces ROS formation, we employed flow cytometry to measure the fluorescence of 2′,7′-dichlorofluorescein (DCF), generated by oxidation of the non-fluorescent 2′,7′-dichlorodihydrofluorescein diacetate (DCFH-DA) after its de-esterification within the cells, in Mv1Lu cells subjected (or not) to statin-mediated CD. As controls, we tested the cellular response to H_2_O_2_-mediated ROS accumulation and/or to N-acetyl-L-cysteine (NAC)-mediated ROS scavenging. Figure 6A shows that CD and H_2_O_2_ induced ROS accumulation to similar degrees, which was blocked by NAC. In an earlier study, we demonstrated that CD induces accumulation of phosphorylated JNK (pJNK) in Mv1Lu cells [16]. To test whether ROS accumulation is involved in JNK activation, cholesterol-depleted cells were treated with NAC, and pJNK formation was measured (Fig. 6B, C). Indeed, NAC treatment significantly reduced the CD-mediated elevation in pJNK. Of note, ROS scavenging also eliminated the CD-mediated increases in LC3-II and p62 (Fig. 6D, E). To test the involvement of JNK activity in the above phenomena, cholesterol-depleted cells were treated with a JNK inhibitor (SP600125), and probed for LC3-II and p62 expression. SP600125 abrogated the accumulation of LC3-II and p62 following CD (Fig. 6F–H). Taken together, the above results suggest that ROS and/or JNK activity is required either for autophagy induction by CD or for the regulation of this process.

**Fig. 7:**
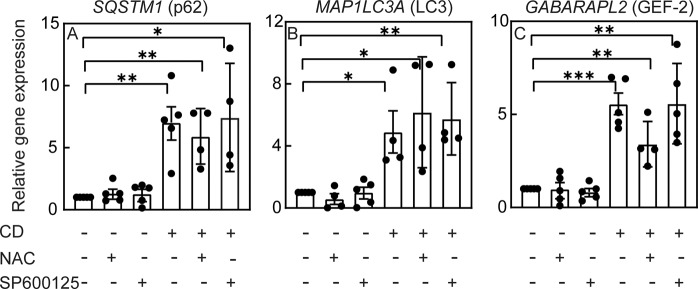
The increased transcription of autophagy markers following cholesterol depletion is independent of ROS and of JNK activity. Mv1Lu cells were treated (or not) for CD by statin (16 h) with or without NAC (10 mM) or SP600125 (20 μM). RT-qPCR was as described under “Materials and methods”. Data (mean ± SEM; 4–5 independent experiments in each case) were normalized to the cDNA levels of β-actin, taking the level of the respective mRNA in untreated cells (control) as 1. The mRNA levels were significantly elevated in cholesterol-depleted cells under all conditions (*P < 0.05; **P < 0.01; ***P < 0.001; Student’s two-tailed *t*-test).

**Fig. 8:**
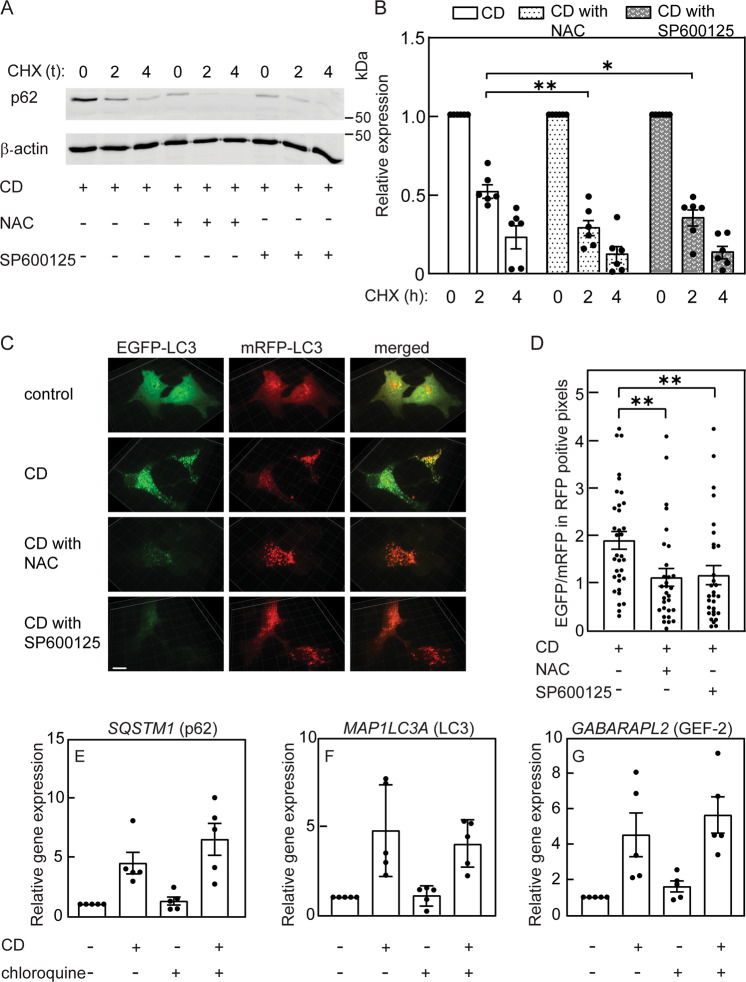
Cholesterol depletion perturbs autophagic flux via ROS and JNK activity. **A**, **B** Mv1Lu cells were subjected to CD by statin (16 h) with or without NAC or SP600125 as in Fig. 6. They were subjected to CHX-chase degradation assay as in Fig. 4E. **A** A representative experiment. **B** Quantification of p62 degradation (mean ± SEM of 6 independent experiments). Data were normalized to β-actin, taking the zero time (prior to CHX addition) for each condition as 1. The degradation rate of p62 was significantly enhanced following NAC or SP600125 treatment (*P < 0.05; **P < 0.01; one-way ANOVA and Dunnett’s multiple comparisons post hoc test). **C** Typical confocal 3-D renditions of LC3-EGFP-mRFP-expressing cells. Mv1Lu cells transiently transfected with the LC3-EGFP-mRFP expression vector ptfLC3 (“Materials and methods”) were subjected (or not) to CD with or without NAC or SP600125 as above, and imaged by spinning disk confocal microscopy. The EGFP signal in untreated cells was mainly diffuse, in accord with low autophagy induction. CD induced a shift to punctate labeling of EGFP and mRFP. Treatment of cholesterol-depleted cells with NAC or SP600125 reduced the EGFP signal, while the increase in mRFP LC3 puncta was retained. **D** Quantification of EGFP relative to mRFP levels in mRFP positive pixels, in single confocal midplanes (see “Materials and methods”). The intensities measured in the respective channels of the confocal microscope were analyzed using SlideBook. Bars are mean ± SEM of measurements on 34 (CD) or 30 (CD with NAC or SP600125) cells. Under the latter conditions, the EGFP/mRFP signal was significantly reduced (**P < 0.01; Student’s two-tailed *t*-test). **E**–**G** Chloroquine treatmen*t* does not induce autophagy-related genes and does not perturb the increase in their expression by CD. Cells were subjected to CD (16 h) with or without chloroquine (10 μM). The mRNA levels of autophagy-related genes were determined by RT-qPCR as in Fig. 7. No significant changes were induced by chloroquine (n.s., *P* > 0.05; Student’s two-tailed *t*-test).

To characterize the effects of NAC or SP600125 on the autophagy-related parameters altered by CD, we measured their effects on the transcript levels of autophagy-related genes (*SQSTM1*, *MAP1LC3A*, and *GABARAPL2*) following CD. ROS scavenging by NAC or JNK inhibition by SP600125 had no significant effect on the increased mRNA levels of all three genes induced by CD (Fig. 7A–C). This suggests that the transcriptional regulation of autophagy by CD is independent of ROS and JNK activity, and that decreased expression of *SQSTM1* mRNA is not the basis for the reduced p62 protein in cholesterol-depleted cells treated with NAC or SP600125. Such a reduction may result from enhanced degradation of p62. This may be mediated by ROS scavenging (e.g., by NAC), or by reduced JNK activity. To test these possibilities, Mv1Lu cells were cholesterol depleted in the presence or absence of NAC or SP600125, and the effects on the degradation rate of p62 were measured in the presence of CHX. NAC or SP600125 accelerated the degradation kinetics of p62 in cholesterol-depleted cells (Fig. 8A, B), suggesting an enhancement of autophagic flux. This was also visualized by confocal microscopy (Fig. 8C, D). Here, cells were transfected with the tandem LC3-EGFP-mRFP vector ptfLC3 [49], subjected (or not; control) to CD, and treated (or not) with NAC or SP600125. The EGFP signal in untreated cells was mainly diffuse, in accord with low autophagy induction. CD induced puncta of both EGFP and mRFP signals, with a high degree of overlap. Treatment of cholesterol-depleted cells with NAC or SP600125 reduced the overall EGFP signal, with little effect on the RFP signal (Fig. 8C), thus reducing the EGFP/mRFP signal in mRFP positive pixels (Fig. 8C, D). This is in accord with localization of LC3-EGFP-mRFP to autolysosomes, as expected in cells with unperturbed autophagic flux. The enhanced degradation of p62 following NAC or SP600125 treatment of cholesterol-depleted cells and the reduction in the EGFP signal of LC3-EGFP-mRFP indicate that ROS and/or JNK activity disrupt autophagic flux prior to autophagosome-lysosome fusion. To test whether perturbation of autophagic flux suffices to induce expression of autophagy-related genes, we treated Mv1Lu cells (cholesterol depleted or not) with chloroquine, which disrupts autophagic flux and induces accumulation of LC3 puncta (Fig. 3G, H). Chloroquine alone did not alter the expression level of autophagy-related genes, and the increased expression of these genes induced by CD persisted in the presence of chloroquine (Fig. 8E–G). Taken together, these results suggest that disruption of autophagic flux by itself is not sufficient for the upregulation of these genes.

## Discussion

Alterations to cholesterol homeostasis, which can occur in several diseases or be induced by statins, have major consequences on cellular function [9–12, 15, 16]. Autophagy is a lysosome-dependent degradation program essential for maintenance and re-establishment of cellular homeostasis [17–21]. CD was reported to induce autophagy [28], and to stimulate SREBP-2-dependent expression of autophagy genes [29]. Moreover, changes of the cholesterol level can regulate autophagosome transport and fusion with the lysosome [30, 31], and recent studies show involvement of a cholesterol transfer protein in autophagosome biogenesis [50, 51]. A potential connection between cholesterol and autophagy may be provided by ROS, which is induced by statin treatment [32, 33], and regulates autophagy by several mechanisms [34, 35, 37–41]. However, the interdependence between CD, enhanced transcription of autophagy genes, induction of autophagy, and ROS accumulation are unclear.

To examine whether mild CD alters the expression pattern of autophagy-related genes, we conducted an RNA-seq analysis comparing the transcriptome of untreated and cholesterol-depleted (~30% depletion) Mv1Lu cells. We observed a highly significant increase in the expression of multiple autophagy-related genes (Figs. 1 and 2), a selection of which was further confirmed by RT-qPCR (Fig. 5). The autophagy genes whose expression was modified by CD were scattered onto multiple steps of the autophagy process (Supplementary Fig. 4), suggesting a mainly positive regulation of autophagy by statin treatment.

To validate that CD induces autophagy in our cells, we measured LC3 expression and lipidation following two different CD treatments (statin inhibition of cholesterol synthesis, and cholesterol absorption by HPβCD) (Fig. 3A–D). Under both conditions, we observed accumulation of LC3-II, indicative of autophagy induction. This conclusion was confirmed by the CD-mediated redistribution of LC3 to a punctate pattern (Fig. 3E–H). In the context of selective autophagy, ubiquitinated cargoes (e.g., damaged organelles or proteins) are recognized by the ubiquitin receptor p62, linking them to the forming autophagosome [24–27]. When autophagy proceeds unperturbed, both p62 and its cargo undergo degradation in the lysosome [23]. Indeed, induction of autophagy by rapamycin treatment significantly reduced p62 protein level (Fig. 4A, B), while leaving its transcript level (*SQSTM1*) unchanged (Fig. 5A). In contrast, CD by statin or HPβCD treatment resulted in p62 accumulation (Fig. 4A–D). Moreover, *SQSTM1* transcript levels were also increased by CD (Figs. 2B and 5A). Given the enhanced *SQSTM1* mRNA expression, the observed increased levels of p62 protein following CD do not preclude its enhanced degradation, which is typical of autophagy [23]. Such a decrease may require compensation by p62 synthesis in order to keep autophagy going. We therefore employed protein synthesis inhibition by CHX to study the effects of CD on the degradation kinetics of p62 (Fig. 4E, F). The enhanced degradation of p62 in cholesterol-depleted cells confirmed the induction of autophagy under these conditions. Of note, the balance between enhanced p62 mRNA expression and the accelerated degradation of p62 protein in cholesterol-depleted cells results in an increase in the level of p62 protein. Of note, previous reports have also identified p62 accumulation under prolonged autophagy-inducing conditions [52, 53].

To test whether there is a dependence between enhanced transcription of autophagy-related genes and autophagy induction by CD, we combined CD with actinomycin D inhibition of transcription. Under these conditions, the hallmarks of autophagy (altered LC3 expression pattern and p62 reduction) were retained (Fig. 5D–F), confirming that autophagy mediated by CD does not require transcription. The notion that autophagy can be ignited without activation of transcriptional programs is supported by the ability of compounds that modify protein acetylation to induce autophagosome formation in cytoplasts (enucleated cells) [54]. However, autophagy can be modulated by transcriptional programs, as multiple autophagy regulators are coordinately regulated with factors involved in lysosomal biogenesis (e.g., by activity of members of the microphthalmia/transcription factor E (MiT/TFE) family), supporting increased autophagic flux [55, 56]. In this context, activation of TFEB-dependent gene expression programs was proposed to result from cholesterol sensing in lysosomes [57]. The importance of lysosome-localized cholesterol in regulating cellular homeostasis is underscored by the cholesterol-dependent regulation of mTOR activity at the lysosome membrane [58]. This latter mechanism provides a potential link between CD and autophagy induction, via mTOR inhibition (*i.e*., emulating the effect of rapamycin). An additional mechanism by which statins can reduce mTOR activity, thus promoting autophagy, was shown in leukemic cells to involve the reduction of activated Akt in cholesterol-rich domains (lipid rafts) [59]. Indeed, CD treatment of Mv1Lu cells altered the expression of multiple genes related to the mTOR signaling pathway (Supplementary Fig. 5), suggesting potential involvement of this pathway in the mediation of the effects of CD.

Statin treatment induces ROS [32, 33] (Fig. 6A). ROS-mediated JNK activation is a well-established signaling axis [60–62]; accordingly, the cholesterol-dependent increase in pJNK was abrogated by NAC treatment (Fig. 6B, C). Moreover, NAC or SP600125 inhibited the accumulation of both LC3-II and p62 (Fig. 6), suggesting involvement of both ROS and JNK activity in autophagy induced by CD. The reduction in p62 may result from inhibition of expression or from enhanced degradation. Our demonstration (Fig. 7A) that *SQSTM1* (p62) mRNA levels are increased by CD also in the presence of NAC or SP600125 suggests that ROS or JNK activity do not inhibit p62 expression. Conversely, NAC or SP600125 enhanced p62 degradation in cholesterol-depleted cells (Fig. 8A), providing a mechanism for p62 reduction. To test for an increase in autophagic flux, we employed confocal microscopy to assay the fluorescence pattern of the tandem LC3-EGFP-mRFP construct (ptfLC3) in cells subjected to CD and treated with NAC or SP600125 (Fig. 8C, D). In this assay, a block in autophagosome maturation into autolysosomes leads to an increase in doubly-labeled red and green (i.e., yellow) pixels. Release from such a block would decrease the green signal following exposure to the acidic pH of the lysosome [23, 49]. Indeed, such a reduction in the green signal (in red pixels) was observed upon treatment of cholesterol-depleted cells with NAC or SP600125. Together with the reduced degradation of p62, these findings suggest that ROS and JNK activity perturb the autophagic flux.

Former studies showed that ROS regulates autophagy through both transcriptional and post-transcriptional mechanisms [37]. Thus, the ROS-responsive transcription factor NRF2 is intertwined with p62 in a positive feedback loop, leading to increased p62 expression [63]. Moreover, ROS-induced calcium release from lysosomes induces autophagy and transcription of lysosome/autophagy-related genes through TFEB, providing an additional ROS-induced autophagy-related transcription program [64]. At the post-transcriptional level, ROS was shown to inhibit ATG4 (a protease involved in deconjugating LC3 from phosphatidyl-ethanolamine), allowing for accumulation of LC3-II on phagophores [35, 65]. Interestingly, ATG4 also regulates autophagosome maturation [66], suggesting a putative mechanism by which this latter step can also be regulated by ROS accumulation. In accord with ROS-induced inhibition of the late steps of autophagy, H_2_O_2_ (a source for increased cellular ROS) blunts lysosome fusion [67]. Furthermore, JNK activation by ROS was shown to induce autophagy in mouse mesenchymal stem cells [68], without accumulation of p62.

In conclusion, the findings reported here indicate that CD has several effects on autophagy induction and regulation, which are at least partially independent: (i) Increased expression of autophagy genes. This specific effect is not essential for autophagy induction, and is not mediated by ROS or JNK activity. (ii) Induction of autophagy. This branch was also independent of ROS or JNK activity. (iii) Attenuation of autophagic flux. This effect, which was apparent by the accumulation of p62 protein due to an increase in its mRNA level and a slower degradation of the protein formed, depended on CD-mediated ROS formation and JNK activation, since it was reversed by NAC or SP600125.

## Materials and methods

All the materials and methods are described in the Supplemental Materials and Methods file.

## Supplementary information

Supplementary files description

Supplemental materials and methods

Supplementary figure legends

Supplementary Figure 1

Supplementary Figure 2

Supplementary Figure 3

Supplementary Figure 4

Supplementary Figure 5

Supplementary Table 1

## Data Availability

Data are presented in the main manuscript or additional supporting files. The RNA-seq data are uploaded to the NCBI Gene Expression Omnibus (GEO) with accession number GSE179869. All other data and materials will be made available by the corresponding authors upon reasonable request.
