# Supplementary material for: Autophagy is induced and modulated by cholesterol depletion through transcription of autophagy-related genes and attenuation of flux
Source: Cell Death Discov. 2021 Oct 29;7:320. doi: 10.1038/s41420-021-00718-3 (PMC8556405; doi:10.1038/s41420-021-00718-3)
Supplement: Supplementary file 1 — Supplementary files description [file 41420_2021_718_MOESM1_ESM.docx]

**SUPPLEMENTARY FILES DESCRIPTION**

1. Supplemental Materials and Methods, including the relevant references. It is a text file (Word docx file).
2. Supplementary Fig. 1. TreeMap of biological processes upregulated following cholesterol depletion (TIFF file).
3. Supplementary Fig. 2. TreeMap of biological processes downregulated following cholesterol depletion (TIFF file).
4. Supplementary Fig. 3. TreeMap of upregulated cellular compartment terms following cholesterol depletion (TIFF file).
5. Supplementary Fig. 4. KEGG graph of the autophagy pathway (TIFF file).
6. Supplementary Fig. 5. KEGG graph of the mTOR pathway (TIFF file).
7. Supplementary Figure Legends (Word docx file).
8. Supplementary Table 1. List of genes altered more than 2-fold by cholesterol depletion (Excel file).
