# Supplementary material for: Autophagy is induced and modulated by cholesterol depletion through transcription of autophagy-related genes and attenuation of flux
Source: Cell Death Discov. 2021 Oct 29;7:320. doi: 10.1038/s41420-021-00718-3 (PMC8556405; doi:10.1038/s41420-021-00718-3)
Supplement: Supplementary file 2 — Supplemental materials and methods [file 41420_2021_718_MOESM2_ESM.docx]

**MATERIALS AND METHODS**

###### Reagents

Hanks' balanced salt solution (HBSS) with Ca^2+^/Mg^2+^, without phenol red; cat. #009015237500), phosphate-buffered saline (10x; cat. #001623237500) and 4-(2-hydroxyethyl)-1-piperazineethanesulfonic acid (HEPES, 1M, pH 7.3; cat#:000773233100) were from Bio-Lab Ltd. (Jerusalem, Israel). Lovastatin (cat. #438185) was obtained from Merck-Calbiochem (Darmstadt, Germany). Fatty acid free bovine serum albumin (BSA) (fraction V; cat. #10-775-835-001) was from Roche Diagnostics (Manheim, Germany). Na_3_VO_4_, mevalonate (as DL-mevalonic acid lactone; cat. #M4667), 2-hydroxypropyl-β-cyclodextrin (HPβCD; cat. #H107), protease inhibitor cocktail (cat. #P8340), phosphatase inhibitor cocktails 2 and 3 (cat. #P5726 and #P0044, respectively), actinomycin D (cat. #A9415), the JNK inhibitor SP600125 (competes with ATP to inhibit c-Jun phosphorylation; cat. #S5567), N-acetylcycteine (NAC; cat. #A8199), rapamycin (cat. #553210), 2’,7’-dichlorodihydrofluorescein diacetate (DCFH-DA; cat. #D6883) and chloroquine (cat. #C6628) were from Sigma-Aldrich (St. Louis, MO). The ptfLC3 expression vector [[1](#_ENREF_1)], encoding LC3 fused to EGFP and mRFP (LC3-EGFP-mRFP), was a gift from Tamotsu Yoshimori (Addgene plasmid #21074). Gel electrophoresis reagents were from Bio-Rad (Hercules, CA). All other reagents were from Sigma.

**Antibodies**

Rabbit antibodies to LC3B (cat. #L7543) were from Sigma-Aldrich. Alexa Fluor (Alexa) 488-goat anti rabbit (GαR) IgG (cat. #R37116) was obtained from Invitrogen-Molecular Probes (Eugene, OR). Normal goat γ-globulin (cat. # 005-000-002), peroxidase-conjugated goat anti-mouse (GαM) IgG (cat. #115-035-062) and peroxidase-GαR IgG (cat. #115-035-144) were from Jackson ImmunoResearch Laboratories (West Grove, PA). Rabbit antibodies to phospho (p) JNK (cat. #9251) and total (t) JNK (cat. #9252) were from Cell Signaling Technology (Danvers, MA). Mouse anti-β-actin (cat. #0869100-CF) was from MP Biomedicals (Solon, OH). Polyclonal rabbit anti-p62/SQSTM1 (cat. #PM045) was from MBL International (Woburn, MA).

**Cell culture**

Mv1Lu mink lung epithelial cells (cat. #CRL-6584) from American Type Culture Collection (ATCC; Manassas, VA) were grown in Dulbecco's modified Eagle's medium (DMEM) as described [[2](#_ENREF_2)]. All media and cell culture reagents were from Biological Industries Beit Haemek (Beit Haemek, Israel). The cells were routinely tested by RT-PCR for mycoplasma contamination.

**Cholesterol depletion**

Mv1Lu cells were subjected to cholesterol depletion (CD) by statin-mediated metabolic inhibition of HMG-CoA reductase as described [[3](#_ENREF_3), [4](#_ENREF_4)]. The cells were incubated (16 h) with 50 μM lovastatin and 50 μM mevalonate in medium supplemented with 10% lipoprotein-deficient fetal calf serum (LPDS), prepared as described earlier [[4-6](#_ENREF_4)]. Mevalonate (the product of HMG-CoA reductase, added to prevent excessive reduction of mevalonate) is added at a level that reduces cholesterol production but is sufficient for farnesylation and geranylgeranylation [[4](#_ENREF_4), [6](#_ENREF_6), [7](#_ENREF_7)]. This treatment reduced the free cholesterol level, which represents almost exclusively membrane cholesterol, by 30-33%, measured by us earlier in the Mv1Lu cells [[4](#_ENREF_4)].We have shown previously that this treatment has no detectable effect on cellular phospholipids or fatty acid composition [[6](#_ENREF_6)]. In some experiments, cholesterol extraction by HPβCD [[8](#_ENREF_8), [9](#_ENREF_9)] was used as an alternative method to reduce membrane cholesterol. HPβCD treatment was conducted basically as described earlier for methyl-β-cyclodextrin [[4](#_ENREF_4), [10](#_ENREF_10)]. Briefly, the medium was replaced by DMEM containing 10% LPDS, and 15 mM HPβCD were added. The cells were incubated for 16 h at 37 °C, as was done for the statin treatment. The reduction in free cholesterol was similar to that in the statin-treated cells [[4](#_ENREF_4)].

**Immunoblotting**

Mv1Lu cells were cultured overnight in 6-well plates, and subjected (or not; control) to CD treatment. Where indicated in the figure legends, cells were treated with actinomycin D (1 µg/ml), NAC (10 mM) or SP600125 (20 μM), which were added at the start of the statin treatment. After 16 h, cells were starved in serum-free medium (2 h, 37°C), followed by lysis on ice (30 min) with lysis buffer [420 mM NaCl, 50 mM Hepes, 5 mM EDTA, 1% NP-40, 3 mM dithiothreitol, protease inhibitor cocktails 1 and 2 and 0.1 mM Na_3_VO_4_]. After low-speed centrifugation to remove nuclei and cell debris, the lysates were subjected to SDS-PAGE (10% or 12.5% polyacrylamide) and immunoblotting as described . The blots were probed (12 h, 4°C) by primary antibodies followed by peroxidase-coupled GαR or GαM IgG (1:5000 for 1 h at 22°C). The bands were visual­ized by enhanced chemiluminescence (ECL) using Clarity ECL substrate (cat. #[1705060](http://www.bio-rad.com/en-us/sku/1705060-clarity-western-ecl-substrate-200-ml), Bio-Rad, Hercules, CA), recorded using ChemiDoc Touch imaging system (Bio-Rad) and quantified by Image Lab software (Bio-Rad).

**Measurement of Intracellular ROS Generation**

Intracellular ROS generation was measured using the dichlorofluorescein (DCF) assay [[11](#_ENREF_11)]. Mv1Lu cells in 6-well plates were subjected (or not) to CD by metabolic inhibition (16 h) with or without the ROS scavenger NAC (10 mM). The cells were treated (or not) with H_2_O_2_ (0.75 mM, 2 h; positive control), and incubated at 37°C (30 min) as described [[12](#_ENREF_12)] with the non-fluorescent cell permeable DCFH-DA, dissolved in dimethyl sulfoxide and diluted to 100 μM in cell growth medium lacking phenol red (supplemented with 10% fetal calf serum or LPDS for untreated and CD-treated cells, respectively) . After washing with fresh medium, the fluorescence of the product of the de-esterified reagent after oxidation by ROS to fluorescent (DCF) was determined by FACS set to 485 nm excitation and 525 nm emission.

**Immunofluorescence of endogenous LC3**

Mv1Lu cells grown on glass covers lips were subjected (or not; control) to CD treatment. After 16 h, cells were fixed with 4% paraformaldehyde, permeabilized with Triton X-100 (0.2% in phosphate buffered saline; 5 min), blocked with goat γ-globulin (200 μg/ml, 30 min, 22°C) in cold HBSS containing 20 mM HEPES (pH 7.4) and 2% BSA (HBSS/HEPES/BSA), and labeled successively (each incubation for 45 min at 22°C in the same buffer) by: (i) rabbit anti-LC3B IgG (3 μg/ml); (ii) Alexa 488-GαR IgG (2μg /ml). Cells were imaged using a motorized spinning disk confocal microscope (Yokogawa CSU-22 confocal head mounted on a Zeiss Axiovert 200M microscope, Carl Zeiss Microscopy GmbH, Jena, Germany) equipped with an Evolve CCD camera (pixel size 0.16 microns; Photometrics, Tucson, AZ). Images were acquired under the command of Slidebook™ (Intelligent Imaging Innovations, Denver, CO), using a 63× oil immersion objective (NA 1.4). For calculation of the coefficient of variation of the fluorescence signal, area of cells from a single confocal mid-plane was manually identified, and the average and standard deviation of the fluorescence signal per cell were calculated with Slidebook^TM^.

**Microscopy experiments based on transfected ptfLC3 (LC3-EGFP-mRFP) vector**

Transient transfection with the tandem LC3-EGFP-mRFP ptfLC3 vector was performed using Lipofectamine® 2000 reagent (cat. #[11-668-019](https://www.fishersci.com/shop/products/invitrogen-lipofectamine-2000-transfection-reagent-2/11668019); Invitrogen, Waltham, MA) according to the manufacturer’s instructions. After 32 h, the cells were subjected (or not; control) to CD treatment for 16 h, fixed with 4% paraformaldehyde, and subjected to spinning disc confocal microscopy and analysis as above, except for the objective employed (100× oil immersion objective, NA 1.45). For quantification of EGFP signal on mRFP positive pixels, mRFP-positive signals of single confocal mid-planes of transfected/treated cells (treatments were identical to described above, with the exception that they were initiated 72 h post-transfection) were identified through intensity-based segmentation. EGFP and mRFP signals in the segmented regions were calculated with Slidebook^TM^.

**Degradation measurements by cycloheximide (CHX) chase**

Mv1Lu cells grown in 6-well plates were subjected (or not; control) to statin-mediated CD treatment (16 h). The cells were then serum-starved (2 h), incubated with 300 μM CHX for 0-6 h at 37°C, lysed, and subjected to SDS-PAGE and immunoblotting.

**Real-time quantitative reverse transcriptase-PCR (RT-qPCR)**

Mv1Lu cells grown in 6-well plates were subjected to CD or left untreated (control). Total RNA was isolated by EZ-RNA (cat. #20-400-100, Biological Industries Beit Haemek), followed by reverse transcription using Verso RT-PCR Kit (cat. #AB-1453-B, Thermo Fisher Scientific, Waltham, MA). RT-qPCR analysis of the mRNA levels of the autophagy-related genes *SQSTM1,* *MAP1LC3* and *GABARAPL2* was done in triplicate using KAPA SYBR FAST ABI Prism qPCR kit (cat. #KK4604, Kapa Biosystems-Roche, Wilmington, MA), and quantified with Applied Biosystems 7300 Real-Time PCR System Software (Thermo Fisher Scientific). Non-template controls (NTC) and quantitative standards (β-actin) were included for calibration. Gene expression values were calculated based on the comparative threshold cycle (C_T_) method [[13](#_ENREF_13)]. The real-time RT-qPCR primers used were: (i) For *SQSTM1* - 5'-CAGAGAAGCCCATGGACAG-3' (forward) and 5'-AGCTGCCTTGTACCCACATC-3' (reverse); (ii) For *MAP1LC3A* - 5'-ACAGCATGGTGAGTGTGTC-3' (forward) and 5'-GGGAGGCGTAGACCATATAGA-3' (reverse); (iii) For *GABARAPL2* - 5'-AAAGGATCCAGCTTCCTTCTG-3' (forward) and 5'- CTCTAAAGCTGTCCCATAGTT-3' (reverse); (iii) For β-actin, 5’-GCCATGGATGACGATATCGC-3’ (forward) and 5’-CATTCCACCATCACACCCT-3’ (reverse).

**mRNA library preparation and sequencing**

RNA extraction was carried out as described under RT-qPCR. Sample concentrations were measured *via* Qubit™ RNA HS Assay Kit (cat. #Q32852, Invitrogen), and quality-tested on an Agilent 2200 TapeStation (Agilent, Santa Clara, CA) using high sensitivity RNA ScreenTape (cat. #5067-5579; Agilent). Libraries were prepared using NEBNext® Poly(A) mRNA Magnetic Isolation Module (cat. #E7490, New England Biolabs, Ipswich, MA) according to the manufacturer’s protocol. Double strand cDNA was amplified by 8 cycle PCR. cDNA was quantified by Qubit™ dsDNA HS Assay Kit (cat. #Q32851; Invitrogen) and quality was verified with TapeStation 2200, using high sensitivity D5000 DNA ScreenTape (cat. #5067-5592; Agilent). One ng cDNA was used for preparation of sequencing libraries using the Nextera XT DNA Sequencing kit (NextSeq 500/550 high output kit v2.5, cat. #20024906, Illumina, San Diego, CA; 75 cycles). Quality and concentration of the DNA preparations were verified as described above, and the samples were analyzed on a NextSeq500 sequencer (Illumina).

Raw sequencing reads were trimmed and filtered using fastp 0.20.1 [[14](#_ENREF_14)], then aligned to mink genome assembly (NNQGG.v01) using STAR 2.7.2a [[15](#_ENREF_15)]. Normalization and differential expression analysis were performed in R 4.0.3 using the package DESeq2 1.30.1 [[16](#_ENREF_16)]. The hypergeometric test was used for enrichment testing. Enriched biological process GO terms with p values, both for up and down-regulated genes, were clustered by Revigo [[17](#_ENREF_17)] according to semantic similarity and visualized in a treemap. Mink GO annotations and dog gene homologs were obtained from Ensembl release 103 [[18](#_ENREF_18)]. Pathway graphs for dog homologs were obtained from KEGG [[19](#_ENREF_19)].

**Statistical analysis**

Statistical significance was analyzed by Prism9 (GraphPad Software, San Diego, CA) using two-tailed Student’s t-test or one-way ANOVA followed by Bonferroni or Dunnett’s post hoc test as described in the figure legends. Data are presented as mean ± SEM, with the number of independent repetitions given in each figure legen d. A *P*‐value lower than 0.05 was considered statistically significant (*, *P* < 0.05; **, *P* < 0.01; ***, *P* < 0.001; ****, *P* < 0.0001).

**REFERENCES**

1. Kimura S, Noda T, Yoshimori T. Dissection of the autophagosome maturation process by a novel reporter protein, tandem fluorescent-tagged LC3. Autophagy. 2007;3:452-60.

2. Shapira KE, Gross A, Ehrlich M, Henis YI. Coated pit-mediated endocytosis of the type I transforming growth factor-β (TGF-β) receptor depends on a di-leucine family signal and is not required for signaling. J Biol Chem. 2012;287:26876-89.

3. Hua X, Sakai J, Brown MS, Goldstein JL. Regulated cleavage of sterol regulatory element binding proteins requires sequences on both sides of the endoplasmic reticulum membrane. J Biol Chem. 1996;271:10379-84.

4. Shapira KE, Ehrlich M, Henis YI. Cholesterol depletion enhances TGF-β Smad signaling by increasing c-Jun expression through a PKR-dependent mechanism. Mol Biol Cell. 2018;29:2494-507.

5. Lin S, Naim HY, Rodriguez AC, Roth MG. Mutations in the middle of the transmembrane domain reverse the polarity of transport of the influenza virus hemagglutinin in MDCK epithelial cells. J Cell Biol. 1998;142:51-7.

6. Shvartsman DE, Gutman O, Tietz A, Henis YI. Cyclodextrins but not compactin inhibit the lateral diffusion of membrane proteins independent of cholesterol. Traffic. 2006;7:917-26.

7. Eisenberg S, Beckett AJ, Prior IA, Dekker FJ, Hedberg C, Waldmann H, et al. Raft protein clustering alters N-Ras membrane interactions and activation pattern. Mol Cell Biol. 2011;31:3938-52.

8. Christian AE, Haynes MP, Phillips MC, Rothblat GH. Use of cyclodextrins for manipulating cellular cholesterol content. J Lipid Res. 1997;38:2264-72.

9. Rosenbaum AI, Maxfield FR. Niemann-Pick type C disease: molecular mechanisms and potential therapeutic approaches. J Neurochem. 2011;116:789-95.

10. Scheiffele P, Roth MG, Simons K. Interaction of influenza virus haemagglutinin with sphingolipid-cholesterol membrane domains *via* its transmembrane domain. EMBO J. 1997;16:5501-8.

11. Wang H, Joseph JA. Quantifying cellular oxidative stress by dichlorofluorescein assay using microplate reader. Free Radic Biol Med. 1999;27:612-6.

12. Goldshmit Y, Erlich S, Pinkas-Kramarski R. Neuregulin rescues PC12-ErbB4 cells from cell death induced by H(2)O(2). Regulation of reactive oxygen species levels by phosphatidylinositol 3-kinase. J Biol Chem. 2001;276:46379-85.

13. Livak KJ, Schmittgen TD. Analysis of relative gene expression data using real-time quantitative PCR and the 2^-ΔΔCT^ Method. Methods. 2001;25:402-8.

14. Chen S, Zhou Y, Chen Y, Gu J. fastp: an ultra-fast all-in-one FASTQ preprocessor. Bioinformatics. 2018;34:i884-i90.

15. Dobin A, Davis CA, Schlesinger F, Drenkow J, Zaleski C, Jha S, et al. STAR: ultrafast universal RNA-seq aligner. Bioinformatics. 2013;29:15-21.

16. Love MI, Huber W, Anders S. Moderated estimation of fold change and dispersion for RNA-seq data with DESeq2. Genome Biol. 2014;15:550.

17. Supek F, Bosnjak M, Skunca N, Smuc T. REVIGO summarizes and visualizes long lists of gene ontology terms. PLoS ONE. 2011;6:e21800.

18. Yates AD, Achuthan P, Akanni W, Allen J, Allen J, Alvarez-Jarreta J, et al. Ensembl 2020. Nucleic Acids Res. 2020;48:D682-D8.

19. Kanehisa M, Furumichi M, Sato Y, Ishiguro-Watanabe M, Tanabe M. KEGG: integrating viruses and cellular organisms. Nucleic Acids Res. 2021;49:D545-D51.
