# Supplementary material for: Autophagy is induced and modulated by cholesterol depletion through transcription of autophagy-related genes and attenuation of flux
Source: Cell Death Discov. 2021 Oct 29;7:320. doi: 10.1038/s41420-021-00718-3 (PMC8556405; doi:10.1038/s41420-021-00718-3)
Supplement: Supplementary file 3 — Supplementary figure legends [file 41420_2021_718_MOESM3_ESM.docx]

**Supplementary Figure Legends**

**Figure S1** **TreeMap of biological processes upregulated following cholesterol depletion.** Treemap of significantly enriched biological process Gene Ontology terms of up-regulated genes, sized according to enrichment significance and clustered by semantic similarity.

**Figure S2** **TreeMap of biological processes downregulated following cholesterol depletion.** Treemap of significantly enriched biological process Gene Ontology terms of down-regulated genes, sized according to enrichment significance and clustered by semantic similarity.

**Figure S3** **TreeMap of upregulated cellular compartment terms following cholesterol depletion.** Treemap of cellular compartments Gene Ontology terms of up-regulated genes, sized according to enrichment significance and clustered by semantic similarity.

**Figure S4** **KEGG graph of the autophagy pathway.** A schematic graph of the animal autophagy pathway, using orthologous dog genes (KEGG cfa04140). Rectangular nodes represent gene products, colored by the log2-transformed fold change values of the cholesterol depleted vs. control differential expression comparison.

**Figure S5** **KEGG graph of the mTOR pathway.** A schematic graph of the mTOR signaling pathway, using orthologous dog genes (KEGG cfa04150). Rectangular nodes represent gene products, colored by the log2-transformed fold change values of the cholesterol depleted vs. control differential expression comparison.
