# Supplementary figures and images for: Autophagy is induced and modulated by cholesterol depletion through transcription of autophagy-related genes and attenuation of flux
Source: Cell Death Discov. 2021 Oct 29;7:320. doi: 10.1038/s41420-021-00718-3 (PMC8556405; doi:10.1038/s41420-021-00718-3)

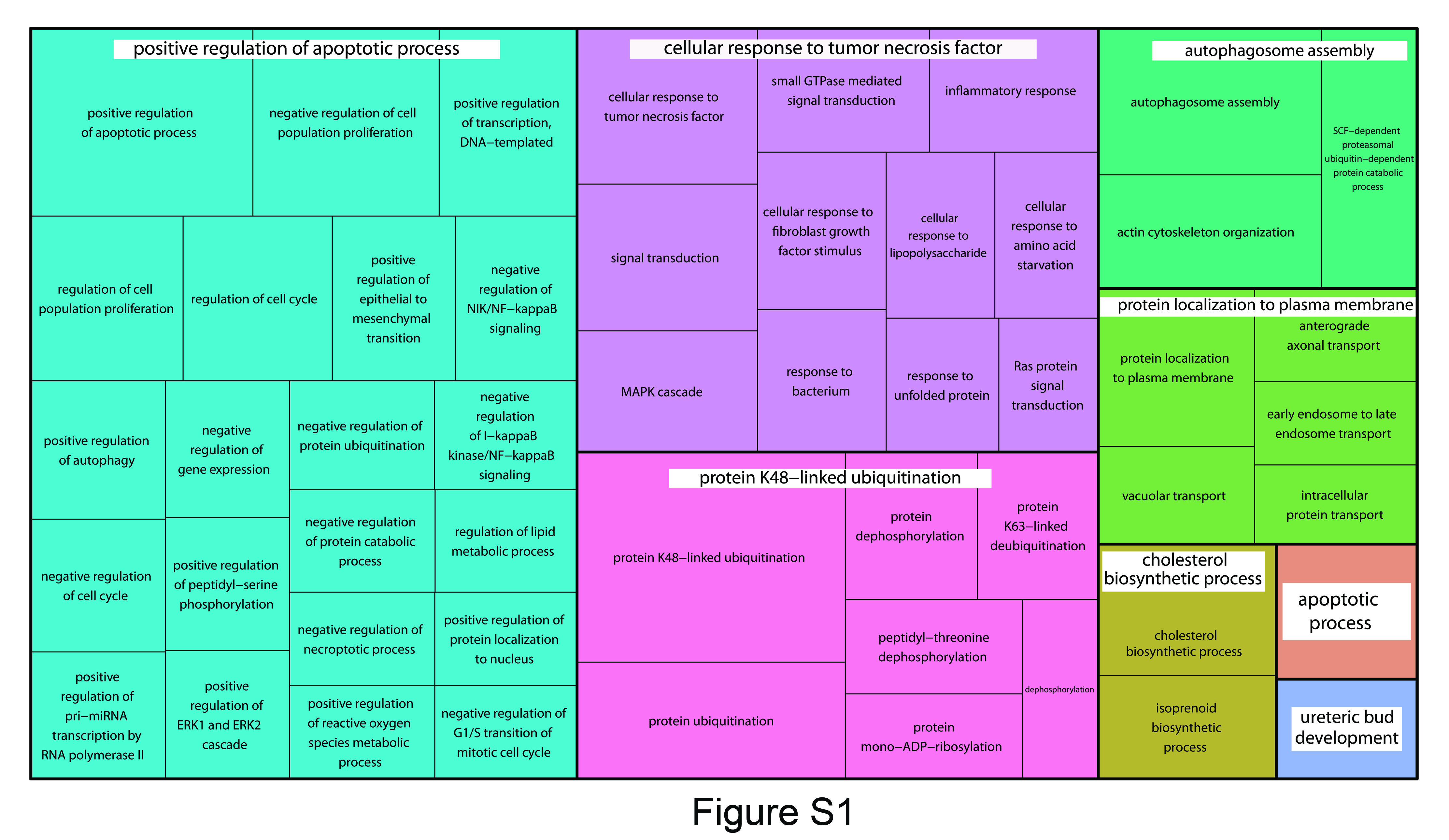

Supplement: Supplementary file 4 — Supplementary Figure 1 [file 41420_2021_718_MOESM4_ESM.tif]

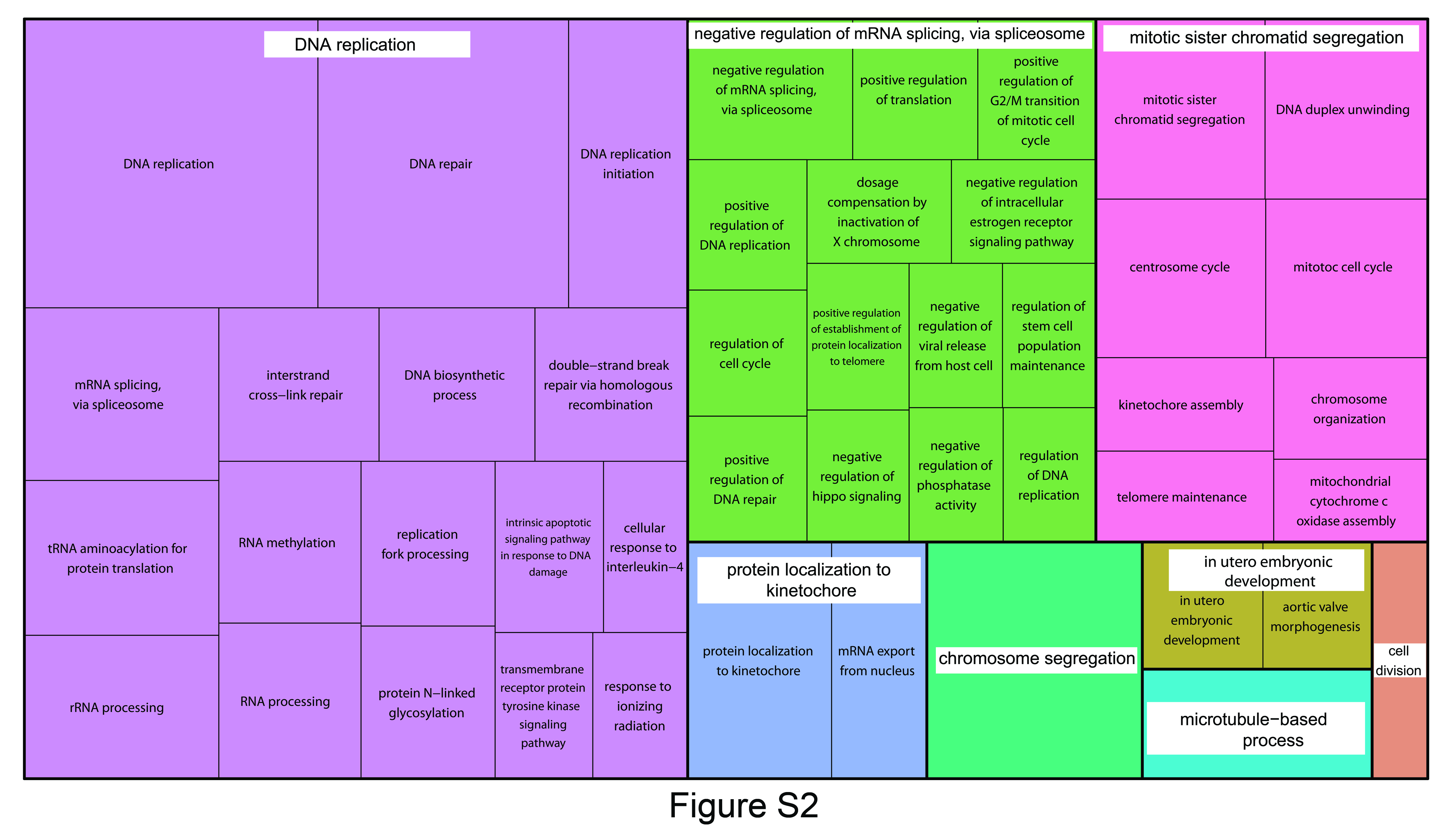

Supplement: Supplementary file 5 — Supplementary Figure 2 [file 41420_2021_718_MOESM5_ESM.tif]

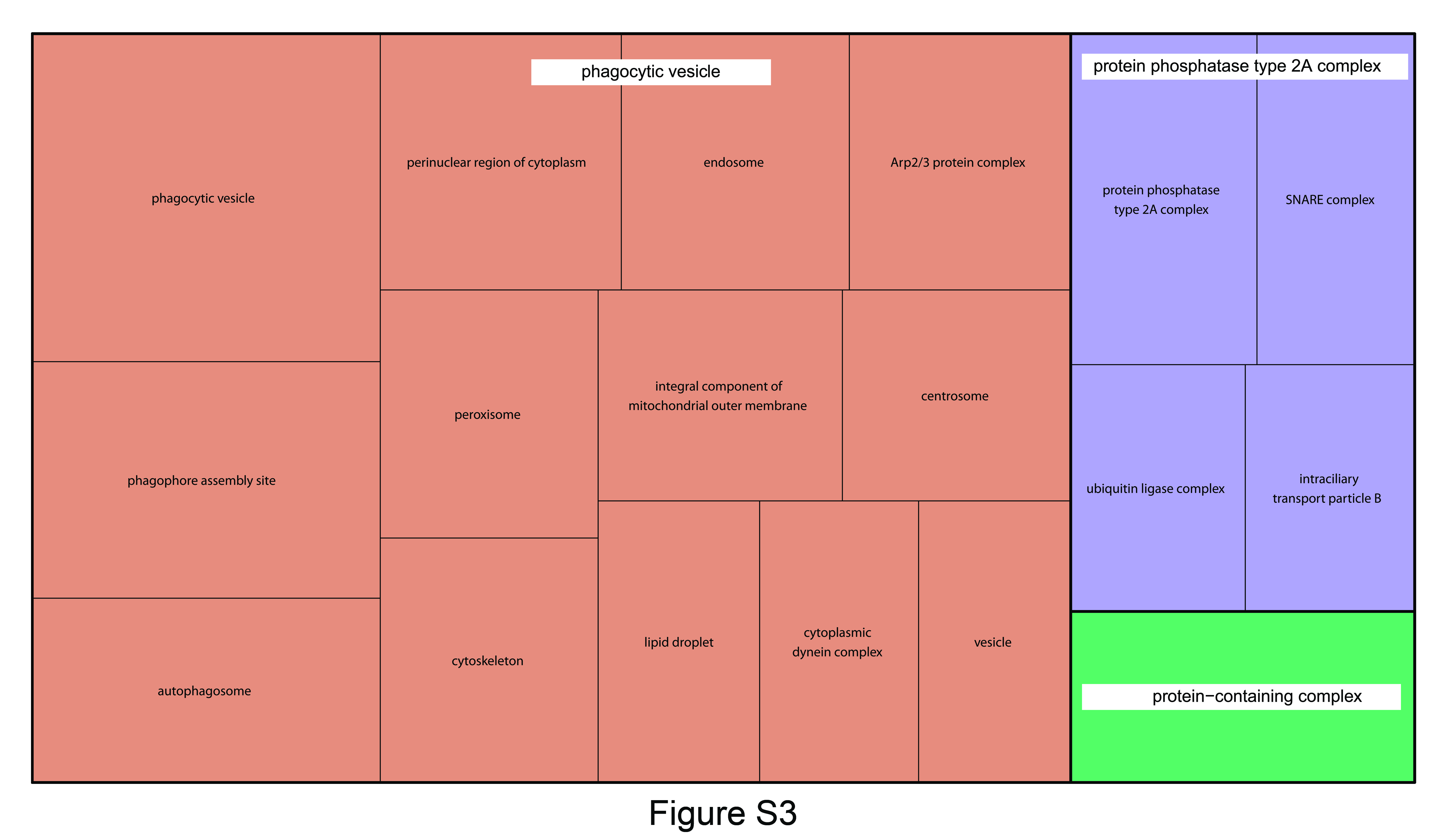

Supplement: Supplementary file 6 — Supplementary Figure 3 [file 41420_2021_718_MOESM6_ESM.tif]

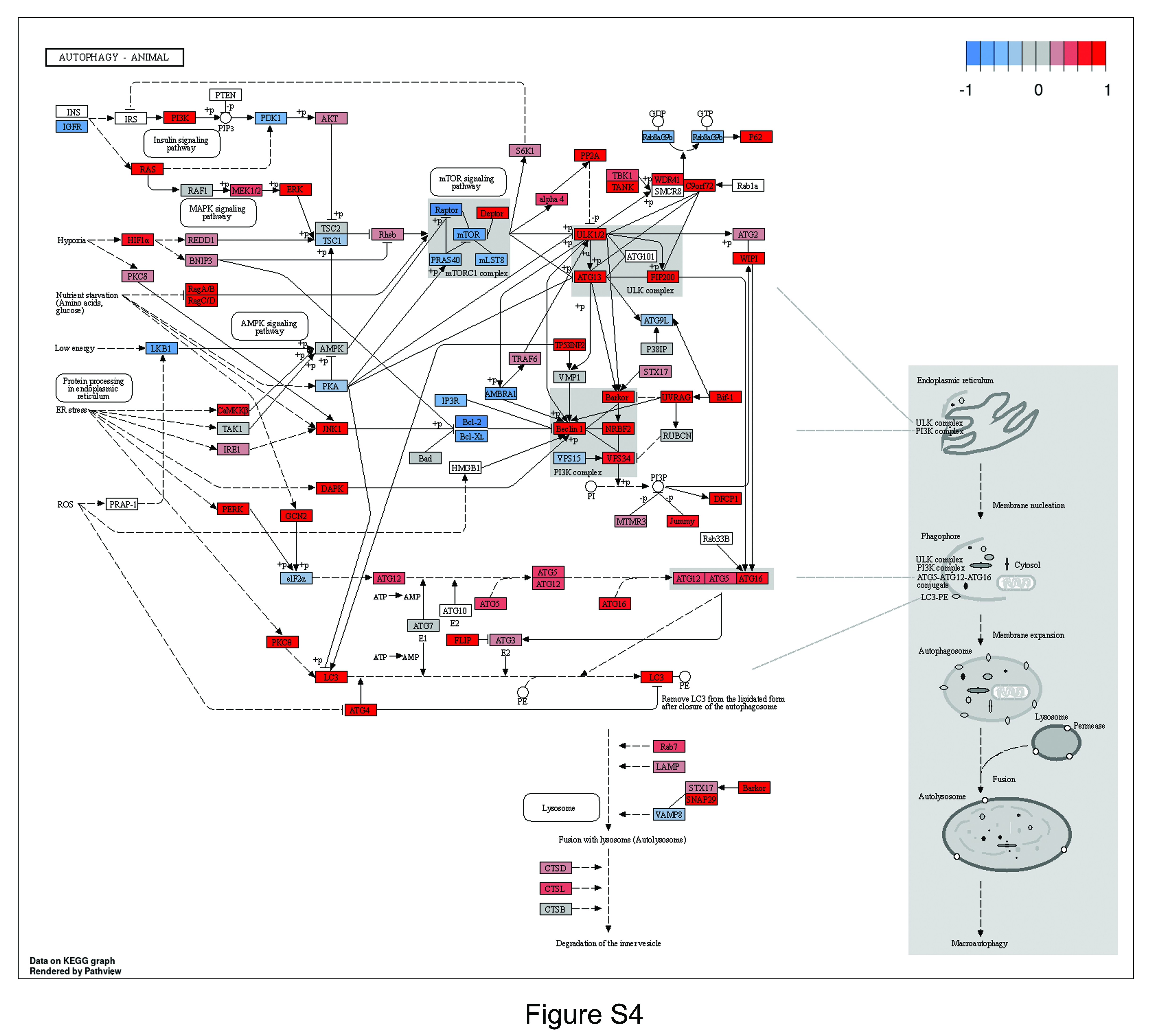

Supplement: Supplementary file 7 — Supplementary Figure 4 [file 41420_2021_718_MOESM7_ESM.tif]

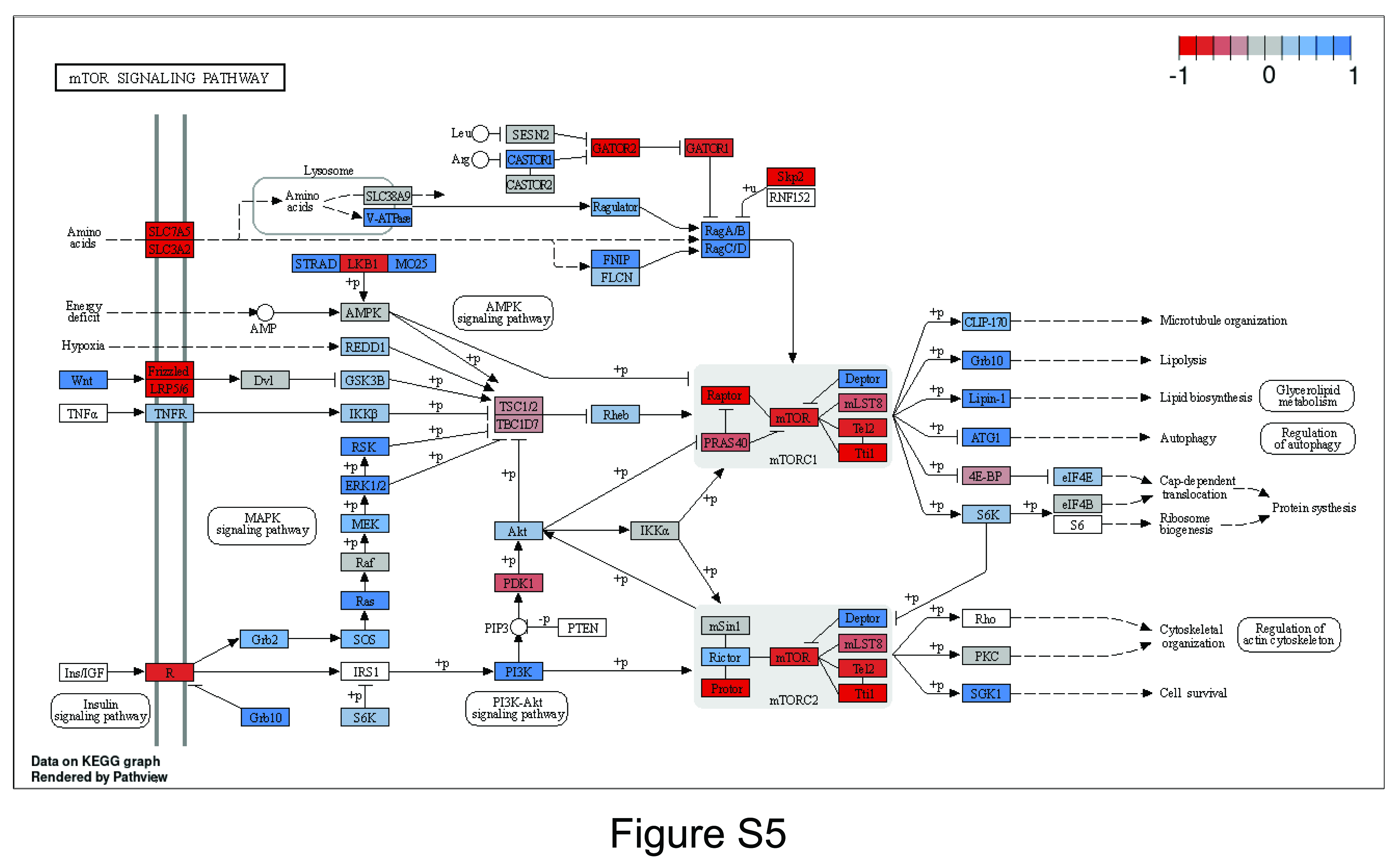

Supplement: Supplementary file 8 — Supplementary Figure 5 [file 41420_2021_718_MOESM8_ESM.tif]
